# Integrating Medical Librarians Into Infectious Disease Rounding Teams: Survey Results From a Pilot Implementation Study

**DOI:** 10.1093/ofid/ofae218

**Published:** 2024-04-17

**Authors:** Mia T Vogel, Lauren H Yaeger, Jason P Burnham

**Affiliations:** Public Health Sciences, Brown School, Washington University in St Louis, St Louis, Missouri, USA; Bernard Becker Medical Library, Washington University in St Louis, St Louis, Missouri, USA; Division of Infectious Diseases, Washington University School of Medicine, St Louis, Missouri, USA

**Keywords:** clinical librarianship, clinical library support, clinical rounding support, decision support, infectious diseases consultation

## Abstract

Medical librarians participating as infectious disease rounding team members add value by facilitating knowledge acquisition and dissemination and by improving clinical decision making. This pilot study implementing medical librarians on infectious disease rounding teams was a well-received and beneficial intervention to study participants.

Studies have highlighted the frequent occurrence of unanswered clinical questions during medical rounds [1, 2]. Unanswered clinical questions can result in delayed or suboptimal patient care, especially in cases involving complex or rare diseases [3]. Medical librarians can help front-line medical practitioners quickly locate and synthesize evidence-based information, helping to address clinical questions that may otherwise go unanswered [3–5]. The inclusion of medical librarians on care teams has been associated with improved patient outcomes, reduced hospitalization duration, and increased satisfaction among healthcare professionals [6–8]. The goal of this pilot study was to determine the need, benefits, feasibility, and acceptability of integrating a medical librarian on infectious diseases (ID) specialty and subspecialty rounding teams for cases where the ID specialty has been deemed a necessary part of the multidisciplinary rounding team.

## METHODS

The Bernard Becker Medical Library at Washington University in St Louis offers medical librarian services, including rounding on clinical teams, which provided a unique opportunity to conduct this study [9].

### Study Design

This was a quasi-experimental, exploratory pilot implementation study designed and interpreted in accordance with guidance available in the literature [10–13]. The evidence-based practice being implemented was reviews of primary medical literature by incorporating a medical librarian into ID specialty service rounding teams. The need, benefits, feasibility, and acceptability of incorporating a medical librarian into ID specialty service rounds were assessed with pre- and postrounding surveys. A librarian rounded with the team and used an iPad and cell phone to conduct literature searches for the team as questions arose, modeled on previous work by Pappas [14]. When possible, the librarian searched in real time at the point of care. For in-depth questions, the librarian returned to her office to complete same-day literature searches and report back to the team.

### Participant Consent Statement

This study was approved by the Washington University in St Louis Institutional Review Board. Participants were providers on the ID medical rounding team. Their completion of the survey confirmed informed consent.

### Survey Development

Pre- and postrounding surveys were developed in accordance with guidance summarized by Ziniel and colleagues [15]. The survey was piloted for language clarity, face validity, and content validity with the study team and experts in ID.

The prerounding survey and postrounding survey each consisted of mostly “yes” or “no” questions and no open-ended questions to ensure the survey could be answered quickly and with minimal participant burden. The prerounding survey assessed the need of the intervention and had questions about whether participants required additional information from the literature, whether they or someone else accessed literature to address identified gaps in knowledge, the extent to which those efforts were successful, and whether they had sufficient time and opportunity in their clinical workflow to review such additional information.

The postrounding survey included questions regarding whether participants were likely to ask the integrated medical librarian for information from the literature (assessing acceptability), whether the librarian provided relevant literature (assessing benefit), the extent to which questions were sufficiently answered (assessing benefit), and whether the integration of a medical librarian on the ID rounding team improved the participant's workflow (assessing fit/feasibility). Feasibility was also defined by successful implementation. Surveys were administered using Qualtrics Software (Qualtrics, Provo, Utah).

### Participant Recruitment

Attending physicians and fellows were included in the study if they were responsible for conducting inpatient clinical service on the weeks the medical librarian was scheduled to join rounds. This pilot spanned October 2019 to March 2020, with the librarian following the same team for 1 full week at a time. The librarian partnered with a clinical fellow leading a rounding team who was recruited by the primary investigator on a Monday and stayed with that team for the week, through Friday. This allowed the librarian to consistently follow the same clinicians and patients, more fully embedding with that team. The prerounding surveys were administered in person on an iPad before starting rounds for the day. The librarian administered the postrounding survey to same team members the same day when rounding ended in person on an iPad or through an emailed link.

### Analysis and Reporting

The pre- and postrounding surveys were analyzed with descriptive statistics using SPSS version 27 software (IBM, Armonk, New York). Absolute and relative frequencies were reported for each question. The results were reported with established guidelines in mind [16, 17]. The manuscript was prepared in accordance with existing guidance on interprofessional team writing [18].

## RESULTS

A total of 41 prerounding surveys and 29 postrounding surveys were initiated. Of those with at least 1 question complete, 40 prerounding surveys (97.6% response rate) and 28 postrounding (68.3% response rate), 100% of pre- and postrounding questions were answered. A total of 1 prerounding and 1 postrounding survey were opened with no questions answered on either.

The structure of the rounding teams was highly variable, but always had only 1 attending, usually 1 fellow (occasionally 2), sometimes 1 medical resident, and 0–4 medical students. The presence of pharmacists was also variable, with an observed maximum of 1 on each team. In some instances, there was a pharmacy student/resident on the team, and in no instances more than 2. The rounding process and duration was highly dependent on the attending physician. Some attendings had sit-down rounds; others rounded outside patient rooms. There were also some hybrids and variations that changed with team structure or clinic schedule.

Almost all preliminary surveys (n = 38 [95.0%]) indicated that faculty and fellows perceived a need for additional or more up-to-date information to answer clinical questions arising during rounds. Respondents stated they accessed the literature after rounding was complete to address any gaps in their knowledge (n = 37 [92.5%]) but indicated they did not have sufficient time to do so (n = 22 [45.0%]) (Table 1).

**Table 1.** Prerounding Survey Questions and Responses

Question	No. (%)	
	Yes	No
During rounds with the ID team, do you ever feel that you need more up-to-date information from the medical literature?	38 (95.0)	2 (5.0)
After rounds with the ID team are over, do you attempt (or have others attempt) to search the medical literature to answer the identified gaps in knowledge?	37 (92.5)	3 (7.5)
If you said “yes” to the previous question, how often are your questions sufficiently answered?		
100%	0 (0.0)	NA
76%–99%	16 (43.2)	
51%–75%	15 (40.5)	
26%–50%	6 (16.2)	
Did your clinical responsibilities/workflow allow time/opportunity to search the medical literature?	22 (55.0)	18 (45.0)

Abbreviations: ID, infectious diseases; NA, not applicable.

The postrounding surveys (Table 2) indicated that participants felt having a librarian on their medical rounding teams improved their ability to find answers to clinical questions and provided them with more satisfying responses. Participants also deemed that the presence of a medical librarian made them more likely to ask for information from the current literature (n = 26 [92.9%]). All responses indicated that the medical librarian followed up to close gaps in knowledge identified during rounding (n = 28 [100%]). Additionally, participants indicated the addition of a medical librarian facilitated integrating literature searches into the workflow (n = 26 [92.9%]).

**Table 2.** Postrounding Survey Questions and Responses

Question	No. (%)	
	Yes	No
While having a librarian on rounds with the ID team, did you feel you were more likely to seek current information from the medical literature?	26 (92.9)	2 (7.1)
After rounds with the ID team were over, did the librarian provide medical literature to answer the identified gaps in knowledge?	28 (100)	0 (0.0)
If you said “yes” to the previous question, how often were your questions sufficiently answered by the librarian?		
100%	9 (32.1)	NA
76%–99%	11 (39.3)	
51%–75%	8 (28.6)	
26%–50%	0 (0.0)	
Did the addition of a medical librarian make finding and using literature fit into your clinical responsibilities/workflow?	26 (92.9)	2 (7.1)

Abbreviations: ID, infectious diseases; NA, not applicable.

## DISCUSSION

The aim of this pilot study was to evaluate the need, feasibility, acceptability, and benefits of incorporating a medical librarian into specialty service ID rounds. Results from the pre- and postrounding surveys indicated the need for and acceptability of the inclusion of a medical librarian on an ID rounding teams at this site. Participants had positive experiences with the addition of medical librarians on the rounding teams and recognized the contributions and value-add of the librarians. The addition of a medical librarian occurred seamlessly, and the medical librarian had the resources, leadership, and infrastructure support required to function effectively, indicating the intervention is feasible. The prerounding survey responses indicated participants perceived a need for additional information to answer certain clinical questions arising on rounds but did not always have time in their workflows to search the medical literature (Table 1). Participants indicated that integration of medical librarians on ID rounding teams made them more likely to access the services of medical librarians and that medical librarians successfully provided relevant literature, demonstrating the effectiveness of the intervention.

A couple of limitations exist in this study. First, while it was smooth to integrate a librarian into medical rounding and the librarian rounded with the team every day during the intervention period, it was difficult to administer the postrounding surveys because team members left at different times or did not have time to take the survey. In these cases, a survey link was emailed the same day but was rarely completed. This may speak to a broader need for postrounding debriefing or huddles. Another limitation is that the survey did not assess whether the inclusion of a medical librarian directly improved patient outcomes; it assessed whether knowledge gaps were addressed. Future research should assess the direct impact of integrating medical librarians on patient outcomes.

In-person rounds were suspended because of the coronavirus disease 2019 pandemic and various related reprioritizations of both physician and librarian resources and efforts, but this program is planned to be continued given its demonstrated feasibility, acceptability, and benefits.

## CONCLUSIONS

Integrating a medical librarian on ID rounds facilitated locating literature to address clinical questions at the point of care, which enhanced continuous learning, physician satisfaction, and clinical decision making. Further research is warranted to better understand the long-term impact of integrating medical librarians with respect to both patient outcomes and healthcare systems, as well as to develop effective strategies for their successful integration into clinical teams.

## Notes

***Acknowledgments.*** The authors thank Dr William Powderly for his support of this work. The authors would also like to thank the librarians, trainees, and faculty who took part in this study.

***Data availability.*** Please submit reasonable data requests to the corresponding author.

***Financial support*.** This study was made possible by National Center for Advancing Translational Sciences (grant number UL1 TR002345, subaward KL2 TR002346), components of the National Institutes of Health, and the National Institutes of Health Roadmap for Medical Research. This publication was also made possible by the National Heart, Lung, and Blood Institute of the NIH (grant number T32 HL130357). This publication was also supported by the Foundation for Barnes-Jewish Hospital and their generous donors and the Washington University Institute of Clinical and Translational Sciences which is, in part, supported by the National Institutes of Health/National Center for Advancing Translational Sciences, Clinical and Translational Science Award (grant number UL1TR002345).
